# Hepatitis C Virus Influences HIV-1 Viral Splicing in Coinfected Patients

**DOI:** 10.3390/jcm9072091

**Published:** 2020-07-03

**Authors:** Paula Martínez-Román, María Rosa López-Huertas, Celia Crespo-Bermejo, Sonia Arca-Lafuente, Isabel Cortegano, Daniel Valle-Millares, María Luisa Gaspar, Luz Martín-Carbonero, Lourdes Domínguez-Domínguez, Pablo Ryan, Ignacio de los Santos, Sara de la Fuente-Moral, Amanda Fernández-Rodríguez, Mayte Coiras, Verónica Briz, on behalf of COVIHEP

**Affiliations:** 1 Laboratory of Reference and Research on Viral Hepatitis, National Center of Microbiology, Institute of Health Carlos III, 28220 Majadahonda, Madrid, Spain; paula.mroman@isciii.es (P.M.-R.); celicres@ucm.es (C.C.-B.); soniarca@ucm.es (S.A.-L.); danielvmillares@gmail.com (D.V.-M.); veronica.briz@isciii.es (V.B.); 2 AIDS Immunopathology Unit, National Center of Microbiology, Institute of Health Carlos III, 28220 Majadahonda, Madrid, Spain; mcoiras@isciii.es; 3 Servicio de Enfermedades Infecciosas, Instituto Ramón y Cajal de Investigación Sanitaria (IRYCIS) and Hospital Universitario Ramón y Cajal, 28034 Madrid, Spain; 4 Department of Immunology, Centro Nacional de Microbiología, Instituto de Salud Carlos III (ISCIII), 28220 Majadahonda, Madrid, Spain; icortegano@externos.isciii.es (I.C.); mlgaspar@isciii.es (M.L.G.); 5 Department of Infectious Diseases, Instituto de Investigación Sanitaria Hospital de la Paz (IdiPAZ), 28046 Madrid, Spain; lmcarbonero@gmail.com; 6 Unidad VIH, Servicio de Medicina Interna, Instituto de Investigación Biomédica del Hospital Doce de Octubre (imas12), 28041 Madrid, Spain; lourdes.dd@outlook.com; 7 Department of Infectious Diseases, Infanta Leonor Hospital, 28031 Madrid, Spain; pabloryan@gmail.com; 8 Servicio de Medicina Interna-Infecciosas, Hospital Universitario de La Princesa, 28006 Madrid, Spain; isantosg@hotmail.com; 9 Servicio de Medicina Interna, Hospital Puerta de Hierro, 28222 Madrid, Spain; sarafm28@hotmail.com

**Keywords:** viral splicing, HIV/HCV, coinfection, HIV reservoir, resting CD4+ T lymphocytes, qPCR, Tat

## Abstract

Coinfection with hepatitis C virus (HCV) influences HIV reservoir size. However, it is unknown whether this coinfection also induces a higher provirus transcription. Viral transcription is promoted by synergy between cellular factors such as NF-κB and the viral regulator Tat. The impact of HCV coinfection on HIV provirus transcription was analyzed in resting (r)CD4 T+ cells (CD3+CD4+CD25-CD69-HLADR-) and rCD4 T cells-depleted PBMCs (rCD4 T- PBMCs) from a multicenter cross-sectional study of 115 cART-treated HIV patients: 42 HIV+/HCV+ coinfected individuals (HIV+/HCV+), 34 HIV+ patients with HCV spontaneous clearance (HIV+/HCV−) and 39 HIV patients (HIV+). Viral transcription was assessed in total RNA through the quantification of unspliced, single spliced, and multiple spliced viral mRNAs by qPCR. Linear correlations between viral reservoir size and viral splicing were determined. A 3-fold increase of multiple spliced transcripts in rCD4 T+ cells of HIV+/HCV+ patients was found compared to HIV+ individuals (*p* < 0.05). As Tat is synthesized by multiple splicing, the levels of Tat were also quantified in these patients. Significant differences in single and multiple spliced transcripts were also observed in rCD4 T- PBMCs. Levels of multiple spliced mRNAs were increased in rCD4 T+ cells isolated from HIV+/HCV+ subjects, which could indicate a higher Tat activity in these cells despite their resting state.

## 1. Introduction

According to the United Nation’s HIV/AIDS program UNAIDS, the human immunodeficiency virus type 1 (HIV) affects 37.9 million people in 2018 and is one of the most prevalent and deadly infections worldwide [1]. To date, finding a cure for HIV remains a challenge due to the early establishment of latent viral reservoirs that are not accessible to suppressive antiretroviral therapy (ART) and are not detectable by the immune system [2,3]. Hepatitis C virus (HCV) infection also remains a major health problem with 71 million chronically infected individuals [4].

Due to shared routes of transmission, up to 6.2% of the HIV-infected individuals are also coinfected with HCV [5]. Coinfection by HIV and HCV alters the natural history of both diseases, worsening the pathogenicity of these viruses and negatively affecting the prognosis [6,7]. Spontaneous HCV clearance occurs in 10–15% of acute infections in HCV-monoinfected individuals but only in less than 10% of HIV-1 infected patients [8]. Our group recently determined that HIV/HCV coinfection enhances the size of HIV proviral DNA in patients exposed to HCV contrasting to HIV monoinfected subjects, in both HCV spontaneous viral clarifiers (HIV+/HCV−) and HCV chronically infected individuals (HIV+/HCV+) [9]. However, it is unknown whether coinfection also affects proviral transcription and if this could significantly affect the strategies for the elimination of HIV-1 reservoir in these subjects.

Splicing of HIV-1 mRNA is a crucial step during viral transcription that is essential for the adequate expression of several viral proteins and their isoforms. Therefore, HIV exploits the cellular splicing machinery for the generation of more than 40 different transcripts [10,11,12]. Unspliced viral mRNA transcripts (~9 kb) code for structural proteins Gag and Pol, single spliced transcripts (~94 kb) code for Vif, Vpu, Vpr, and Env, and multiple spliced transcripts (~2 kb) code for regulatory proteins Nef, Rev, and Tat [13,14,15,16,17,18]. The quantification of spliced mRNAs can help to estimate HIV productive viral infection as increased levels of spliced transcripts reflects an enhanced activity of essential factors involved in gene transcription such as RNA polymerase II [19,20,21]. Moreover, high quantities of inducible multiple spliced HIV RNA may indicate that protein regulator Tat is actively inducing a productive elongation of viral transcript; thereby, increasing proviral transcription and viral particles production [21,22,23].

To our knowledge, there is no information regarding the influence of HCV coinfection on the viral transcription of HIV provirus. We hypothesized that a larger HIV-1 reservoir size in HCV infected individuals may induce an increased HIV-1 transcription from the viral reservoir, likely due to a persistent immune activation. Therefore, changes at viral transcriptional level should be expected between HIV+/HCV−, HIV+/HCV+, and HIV+ patients. Accordingly, in this study we analyzed HIV-1 viral splicing in rCD4 T+ cells and rCD4 T- peripheral blood mononuclear cells (PBMCs) isolated from HIV+ monoinfected patients as well as in HIV+/HCV− and HIV+/HCV+ patients.

## 2. Methods

### 2.1. Patients

We carried out a cross-sectional study of 115 European HIV-1 infected adults under suppressive antiretroviral treatment (ART) selected from five tertiary hospitals in the Community of Madrid (Spain) (Appendix A). They had been under ART for at least 12 months (undetectable viral load) and had maintained CD4 T-lymphocytes values above 500 cells/mm^3^ for at least 12 months before sampling. Exclusion criteria included pregnancy, hepatic decompensation, liver damage caused by alcoholism, active infection with Hepatitis B Virus (HBV), opportunistic infections, substance abuse, and other diseases such as diabetes, nephropathies, autoimmune diseases, hemochromatosis, cryoglobulinemia, primary biliary cirrhosis, Wilson’s disease, α-1-antitrypsin deficiency, and neoplasms. None of the patients had ever taken treatment to HCV infection (conventional treatment based on interferon and ribavirin nor new DAAs).

Evaluation of liver fibrosis was carried out by transient elastography (TE) (FibroScan-502^®^, Echosens, Paris) [24]. IFNL3 rs12979860 single nucleotide polymorphism analysis was determined by qPCR using a custom Taqman polymorphism assay (Life Technologies, Camarillo, CA, USA). Plasma HIV RNA viral load was measured by Amplicor Monitor assay (Roche Diagnostic Systems Inc., Branchburg, NJ, USA) and real-time NASBA (Easy Mag y Nuclisens Easy Q, BioMerieux, Marcy l’Etoile, France) with a detection limit of 20 copies/mL (undetectable viral load). Plasma HCV RNA viral load was measured by COBAS TaqMan HCV Test v2.0 (Roche Diagnostic Systems Inc., Branchburg, NJ, USA). Medical records were used to obtain the following epidemiological and clinical variables: age, weight, height, BMI, Centers for Disease Control and Prevention (CDC) classification system for HIV infection, cART regimens, CD4 and CD8 T cell counts, nadir CD4+, HCV genotype, fibrosis status, and cytomegalovirus (CMV) infection.

Patients were categorized into three groups: (1) HIV+ group: 39 patients infected with HIV-1 and who had not been exposed to HCV (PCR serological analysis: HIV+ and HCV−; negative antibodies for HCV infection: HIV+ and HCV−); (2) HIV+/HCV− group: 34 patients infected with HIV-1 and who had spontaneously clarified HCV infection in the past (PCR serological analysis: HIV+ and HCV−; positive antibodies for both HIV and HCV infection: HIV+ and HCV+; and (3) HIV+/HCV+ group: 42 patients infected with HIV+ and HCV+ (PCR serological analysis: HIV+ and HCV+; positive antibodies for both HIV and HCV infection: HIV+ and HCV+) and naïve to any HCV treatment. The STROME-ID checklist was used to strengthen the design and conduct the study [25].

### 2.2. Ethical Statement

The study protocol was approved by the Research Ethics Committees in all institutions involved [Instituto de Salud Carlos III (CEI PI67_2015-v4), Hospital La Paz (PI-2601), Hospital Universitario 12 de Octubre (16/223), Hospital La Princesa (PI15CIII/00031), Hospital General Universitario Gregorio Marañón (09/2016), Hospital Puerta de Hierro (20.16)]. This study was developed in accordance to the Helsinki Declaration. An informed consent was obtained from each subject, and confidentiality and privacy were assured.

### 2.3. Isolation of PBMCs and Total RNA Extraction

Fifty milliliters of blood samples were taken from all study patients. Briefly, fresh PBMCs were obtained and rCD4+ T cells were purified by negative selection with EasySep™ Human Resting CD4+ T-Cell Isolation Kit (Stemcell Technologies, Vancouver, Canada), as described previously by our group [9]. The purity of CD4+CD25-CD69-HLA/DR-T cells was assessed by flow cytometry as >99%. Total RNA was extracted using AllPrep DNA/RNA Mini Kit (Qiagen, Hilden, Germany) from both rCD4 T+ cells and rCD4 T- PBMCs, following manufacturer’s instructions. RNA was quantified with Nanodrop 1000 (Thermo Fisher Scientific, Waltham, MA, USA).

### 2.4. Quantification of HIV-1 Proviral DNA Integration

The integrated HIV-DNA was measured by nested Alu–HIV-LTR PCR. Briefly, in the first conventional PCR, Alu-1, Alu-2, and LTR L-M667 primers next to Taq DNA polymerase (Roche) were used. Amplicons were quantified through a qPCR (StepOne, Applied Biosystems, CA, USA) using the LTR AA55M, Lambda T primers and MH603 primers next to Taqman Universal PCR Master Mix (Promega). Serial dilutions of 8E5 cell line were used as a standard curve of integrated HIV-DNA, and as a housekeeping gene, CCR5 was used. Values of 2-LTR circle DNA molecules in rCD4 T+ cells and rCD4 T- PBMCs were subtracted from the integrated HIV-DNA value. Information regarding the methodology and procedures involved in the assessment of proviral DNA has been previously described by our group elsewhere [9].

### 2.5. HIV Viral Splicing Analysis by qPCR

HIV viral splicing was analyzed by q-RT-PCR as described previously [5]. Synthesis of cDNA was performed using GoScript™ Reverse Transcription System (Promega, Madison, WI, USA), following manufacturer’s instructions. Briefly, 250 ng of extracted total RNA were diluted in a master mix containing 5X buffer, 25 mM ClMg, dNTPs, RNAsin, and retrotranscriptase, and then incubated at 25 °C for 5 min, 45 °C for 1 h, and 70 °C for 15 min in a C1000 thermal cycler (BioRad, Hercules, CA, USA). Four different qPCRs were carried out in order to measure the different forms of viral splicing forms, using GAPDH as a housekeeping gene. qPCR were performed by adding 2 μL of cDNA to Taqman Universal PCR Master Mix 2X (Applied Biosystems, MA, USA), TaqMan probes FAM/Zen/Iowa Black (Integrated DNA Technologies, Leuven, Belgium), and primers for each specific target were used, as described before with modifications [26]. Primers and Taqman probes used for each target were as follows: unspliced (mf299 primer, mf302 primer, and mf348 TaqMan probe); single spliced (mf222 primer, mf83 primer, and mf226m TaqMan probe modified to 5′-ACCCGACAGGCCCGAAGGAA-3′); multiple spliced (mf84-AK145 primer, mf83 primer, and mf226m TaqMan probe); GAPDH (GAPDH_forward primer, GAPDH_reverse primer, and GAPDH TaqMan probe). PBMCs infected in vitro with NL4-3 HIV strain were used as positive control of active infection (0.2 ng p24/μL). qPCRs were carried out in a StepOne thermal cycler (Applied Biosystems) using a 96-well plate.

### 2.6. Quantification of Tat mRNA Expression

Correlation between level of expression of Tat mRNA and multiple spliced transcripts was also evaluated by q-RT-PCR. cDNA was synthesized from 250 ng of total RNA isolated from patients with different levels of expression of multiple spliced transcripts by using GoScript™ Reverse Transcription System (Promega, Madison, WI, USA), according to manufacturer’s instructions. Different concentrations (100 ng, 25 ng, and 10 ng) of total RNA extracted from PBMCs infected in vitro with NL4-3 HIV strain were used as positive control of an active infection (0.2 ng p24/μL). RNA extracted from uninfected PBMCs was used as a negative control. Quantification of Tat mRNA expression was performed by qPCR using *β-actin* as a housekeeping gene. Five microliters of cDNA were mixed with SYBR^®^ Green Master Mix (Applied Biosystems) and specific primers for each gene: *tat* (Tat_FW primer; Tat_72qpcr RV primer); *β-actin* (b-act s primer; b-act as primer) (Appendix B). Reactions were carried out until 50 cycles in StepOne thermal cycler (Applied Biosystems).

### 2.7. Statistical Analysis

The baseline characteristics of the study population were presented as percentages for categorical variables and as medians and interquartile ranges (IQR) for continuous variables. The normalized status of the different variables included was verified through the Kolmogorov–Smirnov test and the 2-sided Fisher exact test (non-parametric) were used to compare categorical variables between groups. Kruskal–Wallis H test and ANOVA test were performed to compare continuous variables.

Univariate and multivariate analysis with generalized linear models (GLM) and gamma distribution (log-link) were used to evaluate differences in the number of copies of integrated HIV-DNA among study groups. The number of copies is given in mean and standard error of mean (SEM) for each group. This test gives the arithmetic mean ratio (AMR) or the value by which the arithmetic mean of the primary outcome is multiplied. Each multivariate regression test was adjusted by gender, total CD4+T cells, transmission route, AIDS/no AIDS, and HIV-cART.

Analysis of qPCR results was carried out with StepOne Software v2.3 (Applied Biosystems). Amplification results for the different forms of viral splicing were expressed as a fold-change (ΔRQ) with respect to the amplification of the active infection positive control. As every sample was analyzed using duplicates, ΔRQ means and standard errors were calculated for all forms of HIV splicing in every case. Kruskall–Wallis H test was used to compare differences between study groups for the different forms of splicing. Correlation between level of expression of Tat and multiple spliced transcripts was analyzed with Spearman’s correlation test (non-parametric).

Correlations between the different splicing forms and HIV reservoir size were determined with Pearson’s correlation test (parametric).

Data analysis was performed using SPSS v22.0 (SPSS Inc., Chicago, IL, USA). Statistical significance was defined as *p* < 0.05 (2-tailed).

## 3. Results

### 3.1. Study Patients

Patients were categorized into 3 groups according to their HCV status: (1) HIV+ monoinfected group, formed by patients not exposed to HCV; (2) HIV+/HCV− group, whose patients were HIV+ and had been exposed to HCV but experienced HCV spontaneous viral clearance during the first 6 months after HCV infection; and (3) HIV+/HCV+ group, constituted by patients infected with HIV on cART and active HCV−chronic infection naïve to any HCV treatment. Overall, 115 patients (39 HIV+, 34 HIV+/HCV−, and 42 HIV+/HCV+ individuals) were included.

The main socio-demographic characteristics of the study population are shown in Table 1.

All patients were Caucasian, infected with HIV and under ART. Sixty percent (*n* = 69) of patients were male with a median age of 50 years [interquartile range (IQR): 43–54)] and a median HIV infection time of 18.3 years (IQR: 7.8–26.4). Significant differences among the study groups were observed in weight, with a median of 67.40 kg (IQR: 59–79) (*p* < 0.001). Regarding the transmission route, most of the HCV exposed patients contracted the virus by intravenous drug use (IDU) (59.21%, *n* = 44), whereas the prevalent transmission route in HIV+ individuals was sexual (79.50%, *n* = 31) (*p* < 0.001). No significant differences were observed between groups when comparing patients who reached or not AIDS (*p* = 0.067). Regarding HIV treatment, 36.50% (*n* = 42) of patients were receiving ART based on integrase inhibitors and 34.80% (*n* = 40) on non-nucleoside reverse transcriptase inhibitors. In addition, one out of six patients (16.52%; *n* = 19) were receiving simplified therapy (dual therapy or monotherapy). All patients presented a Fiebig stage ≥ V when initiated ART.

Clinical and immunological characteristics of the study population are summarized in Table 2. All patients showed CD4+ T lymphocyte values over 500 cells/µL. Median CD4+ T-lymphocytes were similar among the different study groups (804 cells/mm^3^ (658–1038) for HIV +, 762 cells/mm^3^ (598–938) for HIV+/HCV−, and 729 cells/mm^3^ (632–1040) for HIV+/HCV+ patients, *p* = 0.492)). Median CD8+ counts were also alike (845.50 cells/mm^3^ (539.50–1437.75) for HIV+, 984 cells/mm^3^ (688–1346) for HIV+/HCV−, and 846 cells/mm^3^ (687–1142) for HIV+/HCV+ patients, *p* = 0.769)). No differences among the groups were found in either the CD4:CD8 ratio (1.0 (0.8–1.4) in HIV+, 0.9 (0.7–1.1) in HIV+/HCV−, and 0.9 (0.7–1.1) in HIV+/HCV+ individuals, *p* = 0.087) nor in nadir CD4+ T cells (10.2 cells/mm^3^ (0.8–1.4) in HIV+, 0.9 (0.7–1.1) in HIV+/HCV−, and 0.9 (0.7–1.1) in HIV+/HCV+ subjects, *p* = 0.224).

Clinical characteristics, virological data and genetic data (IL-28 genotype) related to HCV are shown in Table 3. Majority of these individuals were diagnosed with F0–F1 fibrosis stadium (72.36%; *n* = 55) (*p* < 0.001) and were infected with HCV viral genotype 1 (34.21%; *n* = 26) (*p* < 0.001). Lastly, HIV+/HCV− patients had significantly higher (*p* = 0.021) favorable IL-28 genotype (CC) with respect to HIV+/HCV+ individuals supporting the evidence of higher favorable genotype in spontaneously clearance patients.

Finally, proviral HIV-1 DNA size was measured in these subjects (Table 4). A significant increase in proviral HIV-1 DNA size was observed in chronic HIV/HCV-coinfected compared to HIV-monoinfected patients (102.88 ± 18.20 vs. 60.14 ± 11.28, respectively; *p* = 0.042). Similar results were also obtained in spontaneously clarified HCV co-infected patients when compared to HIV-monoinfected individuals (100.60 ± 19.49; *p* = 0.033).

### 3.2. HIV Splicing Analysis

**Figure 1:**
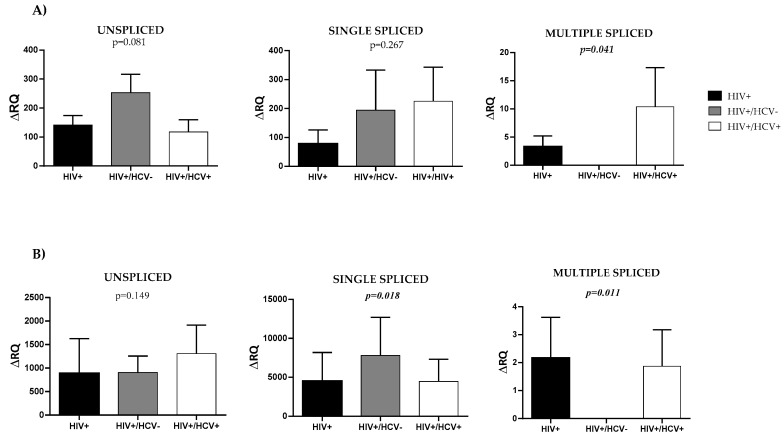
Comparison of the different forms of HIV viral splicing between HIV+, HIV+/HCV− and HIV+/HCV+ patients in (**A**) resting CD4+ T-lymphocytes (CD3+CD4+CD25-CD69-HLA_DR-) and (**B**) rCD4 T- PBMCs. Represented data correspond to ΔRQ mean values determined for each form of splicing in the different groups. Kruskall–Wallis H test was used to compare differences between study groups for the different forms of splicing. Statistical significance was defined as *p* < 0.05 (2-tailed).

HIV splicing analysis revealed significant differences between the three groups of patients in multiple spliced mRNA molecules in rCD4+ T-lymphocytes (Figure 1A), (*p* = 0.041).

The ΔRQ means (±SEM) obtained for these transcripts were 3.31 ± 4.38 for HIV+ group, 0 ± 0 for HIV+/HCV− group and 10.41 ± 0.03 for HIV+/HCV+ group, meaning that coinfected patients showed a 3-fold higher ratio in multiple spliced molecules compared to monoinfected individuals. However, no significant differences were observed in unspliced and single spliced transcripts analysis ((*p* = 0.081) and (*p* = 0.267), respectively). For these, ΔRQ means were 141.45 ± 2.25 and 77.98 ± 6.66 for HIV+ group; 253.12 ± 36.28 and 194.26 ± 36.12 for HIV+/HCV− group; and 117.87 ± 0.44 and 225.43 ± 6.96 for HIV+/HCV+ group.

Amplification results for the different forms of viral splicing were expressed as a fold-change (ΔRQ) with respect to the amplification of the active infection positive control. As every sample was analyzed using duplicates, ΔRQ means and standard errors were calculated for all forms of HIV splicing in every case. Kruskall–Wallis H test was used to compare differences between study groups for the different forms of splicing. Statistical significance was defined as *p* < 0.05 (2-tailed).

In addition, HIV splicing was analyzed in rCD4 T- PBMCs (Figure 1B).

Significant differences were found in single and multiple spliced mRNA transcripts between study groups ((*p* = 0.018) and (*p* = 0.011), respectively). Accordingly, ΔRQ means were 4512.09 ± 4472.15 and 2.16 ± 0.01 for HIV+ group; 7801.52 ± 7401.51 and 0 ± 0 for HIV +/HCV− group; and 4471.34 ± 537.32 and 1.87 ± 0.01 for HIV +/HCV+ group. Single spliced molecules showed a ratio almost 2-fold higher in HIV +/HCV− patients with respect to HIV+ and HIV +/HCV+ individuals. Again, unspliced transcripts analysis did not show significant differences between the three groups of patients (*p* = 0.149) with ΔRQ means 890.61 ± 758.58 for HIV+ group, 906.10 ± 452.01 for HIV +/HCV− group, and 1308.08 ± 2556.69 for HIV +/HCV+ group.

### 3.3. Level Expression of Tat in HIV/HCV Coinfection

No expression of multiple spliced mRNA codifying for Tat was observed by RT-PCR in HCV exposed patients co-infected with HIV even in those showing detection of multiple splice transcripts. However, experiments performed with RNA from in vitro infected PBMCs gave us an idea of the detection limit of this test and the correlation between the detection of Tat and the number of multiple transcripts from a sample.

**Figure 2:**
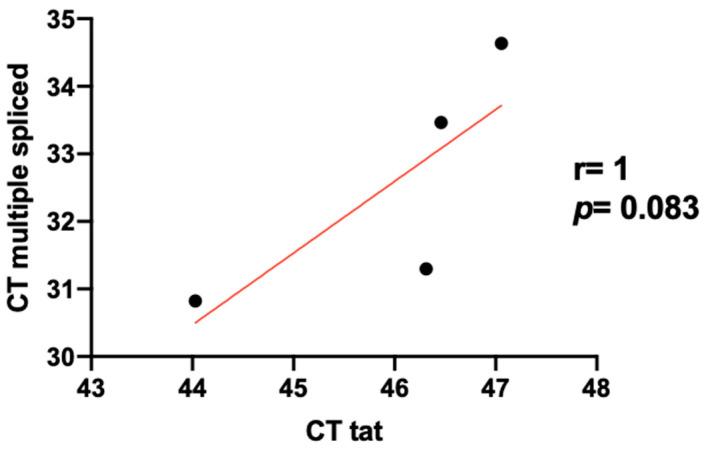
Pearson’s correlation between level of expression of Tat and multiple spliced transcripts. Expression of Tat mRNA level in PBMCs infected in vitro with HIV NL4.3 strain (0.2 μg p24/μL) was analyzed by qPCR. Non-infected PBMCs were used as a negative control and β-actin was used as a housekeeping gene.

The detection limit of the assay was 25 ng of total RNA extracted from PBMCs infected in vitro with NL4–3 HIV strain, which corresponded with 0.2 ng of HIV particles per μL of infection supernatant. With respect to Tat quantification, melting temperature of the fragments was consistently 82.6 °C. Mean C_T_ values were 45.17 for 100 ng of infected PBMCs RNA and 46.75 for 25 ng of the same sample. Relative quantification ratio was determined, where Ratio_(test/calibrator)_ = 2^[(Mean C^_T_^calibrator) – (Mean C^_T_^test)]^ and 100 ng is the amount of sample selected as calibrator, Tat expression was ~3-fold lower (ratio = 0.327) when using 25 ng of RNA. Moreover, regarding the correlation between level of expression of Tat and multiple spliced transcripts, mean C_T_ values of multiple spliced transcripts were 31.06 for 100 ng of HIV infected PBMCs RNA, and 34.04 for 25 ng. Here, multiple spliced transcripts expression was ~8-fold lower (ratio = 0.126) when using 25 ng of RNA. Figure 2 shows a positive correlation between Tat and multiple spliced transcripts expression (r = 1 and *p* = 0.083).

### 3.4. HIV Reservoir Size and HIV Splicing Correlation

**Figure 3:**
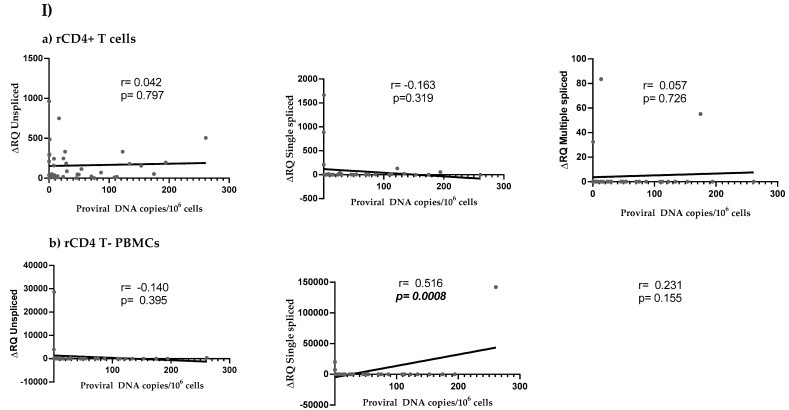

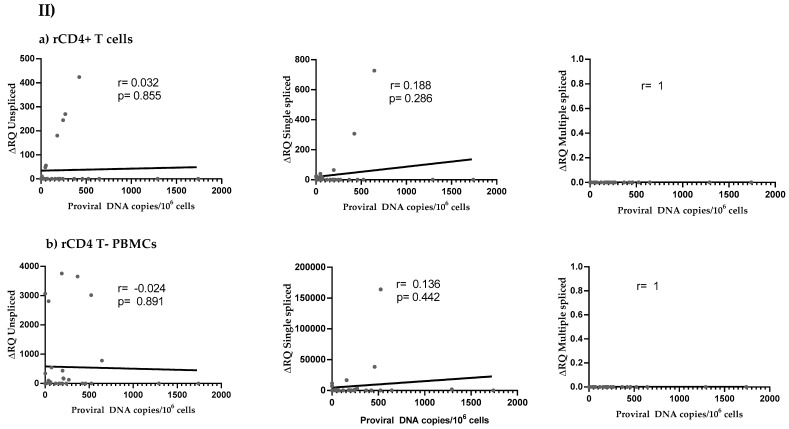

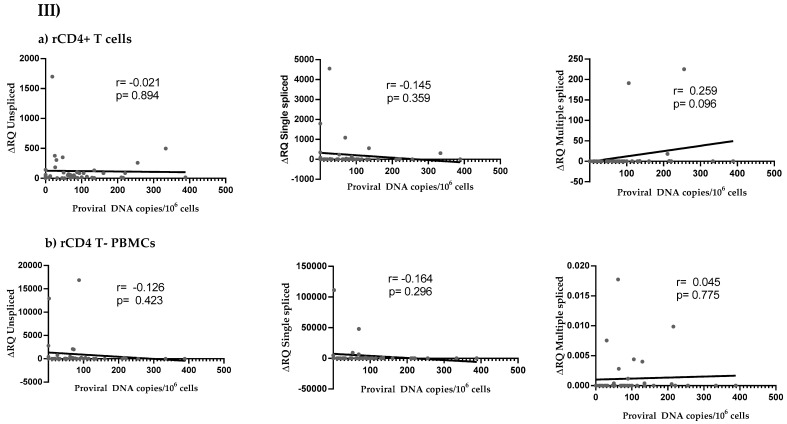
Correlations between HIV reservoir size and different forms of HIV splicing in (**a**) rCD4+ T cells and (**b**) rCD4 T- PBMCs according to the study population: (**I**) HIV+ group, (**II**) HIV +/HCV− group, and (**III**) HIV +/HCV+ group.

Linear correlations between HIV reservoir size (expressed as number of integrated HIV proviral DNA copies per million cells) and HIV viral splicing (expressed as ΔRQ) were assessed (Figure 3). Positive correlations were observed in all study groups for some HIV splicing forms, mostly single and multiple spliced transcripts.

## 4. Discussion

HIV+ individuals are commonly infected with HCV [5] and co-infection alters the pathogenesis of both viruses [6,7]. Our group recently described that HIV reservoir is increased in patients co-infected with HIV and HCV [9]. A similar effect has been previously observed in HIV+ patients co-infected with other viruses such as CMV [27]. Therefore, in this study we analyzed how HCV co-infection may influence HIV proviral transcription and splicing in co-infected patients. Overall, data showed an increase in HIV-1 mRNA transcription in multiple spliced transcripts for rCD4 T-cells, and in both single and multiple spliced transcripts for rCD4 T- PBMCs; probably induced by the continuous activation of these cells due to HCV replication [28]. Multiple spliced mRNAs are a better surrogate marker for the replication-competent reservoir than unspliced mRNAs as splicing requires the presence of several cis-acting sequences in HIV-1 genome. Therefore, the presence of multiple spliced mRNAs reduces the possibility of detecting proviruses with large deletions [29,30]. After cART initiation, the decrease in mRNAs with multiple splicing is more significant than in unspliced mRNAs [31,32,33,34,35,36]. This suggests that HIV-infected cells expressing multiple spliced mRNA may be a more proximal surrogate of cells containing competent proviruses that are reactivated from latency, at least to some extent [29,37].

In our study, the expression of single and multiple spliced mRNA transcripts were enhanced in rCD4+ T cells from HIV+ patients on cART with active HCV−chronic infection in comparison with HIV-1 monoinfected patients. Ongoing production of HIV-1 particles from latently infected cells will produce both unspliced and spliced RNA [38]. Multiply spliced RNA is critical for the production of Tat, Nef, and Rev, which are viral proteins required for efficient production of full-length unspliced RNA [33,38]. In fact, Tat is essential for viral transactivation through the control of viral efficient transcriptional elongation and splicing and its absence impedes proviral transcription [14,39]. Therefore, the enhanced expression of single and multiple spliced HIV-1 transcripts in HIV/HCV chronically co-infected patients may account for HIV-1 reactivation from latency that finally would result in the expression of unspliced transcripts and subsequent complete viral replication from competent proviruses. Following cART initiation in patients with chronic infection, unspliced mRNAs persist in approximately 70–80% of patients [32,38,40,41], which would be in accordance with our results. However, the level of unspliced transcripts were unaltered in HIV-infected patients on cART with active HCV-chronic infection in comparison with HIV-monoinfected patients, suggesting that HIV-1 reservoir is partially reactivated in co-infected patients in spite of the initiation of transcription. The analysis of HIV-1 mRNA transcription and splicing in HIV/HCV co-infected patients that experienced spontaneous clearance of HCV infection showed a different scenario. In these patients, HIV-1 proviral reservoir was reduced in comparison with HIV-infected patients with chronic HCV co-infection and almost the same than in HIV-1 monoinfected patients, as described before [9]. Accordingly, single spliced and unspliced mRNAs were only slightly enhanced in both rCD4 T+ cells and rCD4 T- PBMCs of this group of patients, suggesting that the presence of a more robust and active immune system, which allowed HCV clarification [42,43,44,45], was also interfering with HIV-1 proviral reactivation.

Multiple spliced transcripts are usually less abundant than unspliced RNAs in HIV-infected individuals, both cART-treated or not [29,45,46] and they are difficult to detect in long-term treated HIV-infected patients, unless cells are stimulated ex vivo [29]. Multiply spliced mRNAs include those expressing Tat, and it has been described that suboptimal levels of Tat or its defective function is supposed to facilitate the maintenance of HIV latency [47,48,49,50,51]. Accordingly, mRNAs coding for Tat could not be detected in HIV/HCV coinfected patients, even in those patients with detectable multiple splice transcripts, suggesting that the levels of Tat are below the threshold of detection of our method. In fact, although active viral transcription and splicing were observed, plasma viremia was undetectable in all patients who participated in this study. Furthermore, a higher unspliced/multiple spliced mRNA ratio has been suggested to reflect the higher frequency of HIV-infected cells in the later stages of the viral replication cycle, which is characterized by expression of viral structural proteins [29].

Our study presents other limitations and arises future perspectives. HIV-1 defective proviruses are a major part of the viral reservoir that persists because it cannot be transcribed to functional mRNAs [52]. Even when CD4+ T cell activation is maximal, the transcription of intact proviruses (non-defective) is a stochastic process, meaning that not all viable proviruses are always accessible [53]. Therefore, the quantification of transcriptional initiation from latent HIV-1 proviruses may be overestimating the amount of competitive replicative viruses. Other techniques such as quantitative viral outgrowth assays (QVOA) [54] are more accurate to determine the frequency of the number of cells infected with replicative proviruses but unfortunately could not be performed in our study due to sample volume limitations. Furthermore, it has been observed that HIV-1 provirus preferentially integrates into genes involved in immune activation, allowing the association between early gene expression and CD4+ T cell activation, which consequently increases the probability for HIV-1 provirus to be eventually reactivated and initiate viral transcription [55,56,57]. Because co-infection with HCV induces higher CD4+ T cell activation in HIV patients [28], it would be expected that HIV-1 proviruses integrate into sites that are preferentially transcribed in HIV/HCV co-infected individuals, which would be worth analyzing.

In conclusion, levels of multiple spliced mRNAs were increased in rCD4 T cells isolated from HIV +/HCV+ subjects. This study improves our understanding about the role of HCV in HIV+ co-infected patients during the natural history of co-infection.

## Figures and Tables

**Table 1:** Socio-demographic characteristics and HIV therapy of study patients.

	Total (*n* = 115)	HIV+ (*n* = 39)	HIV+/HCV− (*n* = 34)	HIV+/HCV+ (*n* = 42)	*p*
**Male,***n* (%)	69 (60)	21 (53.8)	25 (73.5)	23 (54.8)	0.158
**Median Age,** years [median (IQR)]	50 (43–54)	47 (39–55)	51 (43–54.25)	50 (45–54)	0.271
**Height,** cm [median (IQR)]	165 (158–174)	164 (154–174)	168 (160–175)	164 (160–174)	0.363
**Weight,** kg [median (IQR)]	67.40 (59–79)	71.50 (61–81)	77.30 (65–84)	60 (54–70)	0.000
**Transmission route,***n* (%)					0.000
IDUs	44 (38.3)	0 (0)	20 (58.8)	24 (57.1)	
MSM	30 (26.1)	15 (38.5)	9 (26.5)	6 (14.3)	
MSW	22 (19.1)	16 (41)	3 (8.8)	3 (7.1)	
Others	19 (16.5)	8 (20.6)	2 (5.8)	9 (21.5)	
**Time of HIV infection,** years [median (IQR)]	18.3 (7.8–26.4)	14.8 (5.7–25.3)	20.5 (4.4–26.7)	19.5 (14.6–28.2)	0.102
**CDC category**, *n* (%)					0.079
A	60 (52.8)	22 (58.4)	18 (52.9)	20 (47.6)	
B	17 (14.8)	5 (12.9)	4 (11.7)	8 (19.1)	
C	28 (24.6)	5 (12.9)	10 (29.5)	13 (30.9)	
Unknown	9 (7.8)	6 (15.4)	2 (5.9)	1 (2.4)	
ALT	26.5 (21.4–37.5)	24.5 (20.0–33.5)	23.0 (19.0–26.0)	38.0 (29.0–49.0)	0.000
AST	29.0 (21.5–46.5)	29.5 (22.3–43.8)	22.0 (12.0–26.6)	46.0 (31.0–60.5)	0.000
CMV					0.671
Yes	43 (37.4%)	12 (30.8%)	14 (41.2%)	17 (40.5%)	
No	13 (11.3%)	5 (12.8%)	5 (14.7%)	3 (7.1%)	
Unknown	59 (51.3%)	22 (56.4%)	15 (44.1%)	22 (52.4%)	

*Notes:* HIV, human immunodeficiency virus; HCV, hepatitis C virus; IDUs, intravenous drug users; MSM, men who have sex with men; MSW, men who have sex with women; CDC, Centers for Disease Control and Prevention classification system for HIV infection; ALT, alanine aminotransferase; AST, aspartate aminotransferase, CMV, cytomegalovirus; %, percentage; and IQR, interquartile range.

**Table 2:** Clinical and immunological characteristics of study patients.

	Total (*n* = 115)	HIV+ (*n* = 39)	HIV+/HCV− (*n* = 34)	HIV+/HCV+ (*n* = 42)	*p*
**CD4+ T-lymphocytes,** cells/µL [median (IQR)]	763 (636–1004)	804 (658–1038)	762 (597–938)	729 (632–1040)	0.492
**CD4+ T-lymphocytes, %** [median (IQR)]	36 (31–42)	38 (32–44)	35 (31–38)	35.83 (31–42)	0.299
**Nadir CD4,** cells/µL [median (IQR)]	240 (170–338)	268 (191–407)	210 (85–299)	254 (171–336)	0.224
**Nadir CD4 %** [median (IQR)]	18 (10–28)	24 (17–30)	13 (6–19)	21 (13–31)	0.001
**CD8+ T-lymphocytes,** cells/µL [median (IQR)]	863 (639.5–1234)	845 (539–1437)	984 (688–1346)	846 (687–1142)	0.769
**CD8+ T-lymphocytes, %** [median (IQR)]	42 (35.50–45)	28 (21–46)	41 (37–45)	42.50 (41–47)	0.090
**CD4:CD8 Ratio**	0.9 (0.7–1.1)	1.0 (0.8–1.4)	0.9 (0.7–1.1)	0.9 (0.7–1.1)	0.087
**ART regimen, *n* (%)**					0.889
**NNRTIs**	40 (34.8)	11 (28.8)	14 (41.2)	15 (35.7)	
**NRTIs**	1 (0.9)	0 (0)	1 (2.9)	0 (0)	
**PIs**	12 (10.4)	3 (7.7)	5 (14.7)	4 (9.5)	
**INIs**	42 (36.5)	17 (43.6)	10 (29.4)	15 (35.7)	
**Dual Therapy**	14 (12.2)	5 (12.8)	3 (8.8)	6 (14.3)	
**Monotherapy**	5 (4.3)	2 (5.1)	1 (2.9)	2 (4.8)	
**Unknown**	1 (0.9)	1 (0.9)	0 (0)	0 (0)	

Notes: HIV, human immunodeficiency virus; HCV, hepatitis C virus; ART, antiretroviral therapy; NRTIs, nucleoside analogue reverse transcriptase inhibitors; NNRTIs, non-nucleoside reverse transcriptase inhibitors; PIs, protease inhibitors; INIs, integrase inhibitors; %, percentage; and IQR, interquartile range.

**Table 3:** Clinical characteristics, virological data and genetic data of study patients related to HCV infection.

	Total (*n* = 115)	HIV+/HCV− (*n* = 34)	HIV+/HCV+ (*n* = 42)	*p*
**Fibrosis,***n* (%)				0.000
F0–F1 (< 6 kPA)	55 (72.4)	23 (67.6)	29 (69)	
F2 (6–9 kPa)	7 (6.1)	0 (0)	7 (16.7)	
F3 (> 9–12 kPa)	3 (2.6)	0 (0)	3 (7.1)	
F4 (> 12 kPa)	1 (0.9)	0 (0)	1 (2.4)	
Unknown	13 (17.1)	11 (32.4)	2 (4.8)	
**HCV genotype, ***n* (%)				0.000
1	26 (34.2)	1 (2.9)	25 (59.5)	
2	2 (2.6)	0 (0)	2 (4.8)	
3	4 (5.26)	0 (0)	4 (9.5)	
4	11 (14.5)	2 (5.9)	8 (19)	
Unknown	34 (44.7)	31 (91.2)	3 (7.1)	
**IL-28 genotype,***n* (%)				0.021
CC	37 (48.7)	23 (67.6)	14 (33.3)	
CT/TC	30 (39.5)	9 (26.5)	21 (50)	
TT	6 (7.9)	2 (5.9)	4 (9.5)	
Unknown	3 (3.9)	0 (0)	3 (7.1)	

Notes: HIV, human immunodeficiency virus; HCV, hepatitis C virus; %, percentage; and kPa: kilopascals.

**Table 4:** Differences in the number of copies of integrated HIV-DNA among study groups.

Groups	Primary Outcome Mean ± SEM	Univariate Analysis ARM (95% CI)	*p*	Multivariate Analysis aAMR (95% CI)	*p*
**HIV+**	60.14 ± 11.28	0	-	0	-
**HIV+/HCV−**	100.60 ± 19.49	1.673 (0.986; 2.838)	0.056	1.815 (1.050; 3.138)	0.033
**HIV+/HCV+**	102.88 ± 18.20	1.711 (1.032; 2.836)	0.037	1.684 (1.018; 2.785)	0.042

Note: Linear regression analysis by generalized linear model (GLM): Univariate analysis comparing HIV-1 DNA provirus vs. HIV+/HCV− group or HIV+/HCV+ groups, respectively; multivariate analysis comparing HIV-1 DNA provirus vs. HIV+/HCV− group or HIV+/HCV+ groups, respectively, after adjustment by CD4+T cells, transmission route, gender, cART, and AIDS/no AIDS. SEM: standard error of the mean; 95%CI: confidence Interval; ARM: Arithmetic mean ratio; and aARM: adjusted arithmetic mean ratio.

## Appendix A

Multidisciplinary group of HIV/Hepatitis viral coinfection (COVIHEP) [In alphabetical order of institutions and authors within each institution]:

- Hospital Universitario 12 de octubre (Madrid-Spain): Laura Bermejo-Plaza; Otilia Bisbal; Lourdes Domínguez-Domínguez; María Lagarde; Mariano Matarranz; Federico Pulido; Rafa Rubio; Mireia Santacreu.
- Hospital Universitario Infanta Leonor (Madrid-Spain): Guillermo Cuevas; Victorino Diez-Viñas; Pablo Ryan; Jesús Troya.
- Hospital Universitario La Paz (Madrid-Spain): Juan Miguel Castro-Álvarez; Marta Gálvez-Charro; Luz Martín-Carbonero; Mario Mayoral-Muñoz.
- Hospital Universitario La Princesa (Madrid-Spain): Ignacio de los Santos; Lucio García-Fraile; Jesús Sanz-Sanz.
- Hospital Universitario Puerta del Hierro Majadahonda (Madrid-Spain): Alfonso Ángel-Moreno; Sara de la Fuente Moral.
- Instituto de salud Carlos III Majadahonda (Madrid-Spain): José Alcamí; Sonia Arca-Lafuente; Verónica Briz, Oscar Brochado; Celia Crespo-Bermejo; Mayte Coiras; Alicia Gómez-Sanz, Amanda Fernández-Rodríguez; Irene Mate-Cano; Paula Martínez-Román; Elena Mateo; Salvado Resino; Marta Sánchez-Carrillo; Daniel Valle-Miralles.
- Research Institute for Medicines (Lisboa-Portugal): Claudia Palladino; Nuno Taveira
- The Roslin Institute (Edinburgh-UK) and CENIDCI, Centro Nacional de Investigación y Desarrollo del Cerdo Ibérico of INIA (Madrid-Spain): María Muñoz-Muñoz.

## Appendix B

Primers used for the analysis of HIV Tat and β-actin:

**PRIMER NAME**	**5’-3’ PRIMER SEQUENCE**	**ORIENTATION**	**PURPOSE**
**Tat FW**	ATGGAGCCAGTAGATCCTA	fwd	Tat
**Tat 72qpcr RV**	AGACAGCGACGAAGACCTC	rev	Tat
**b-act s**	AGGCCCAGAGCAAGAGAGGCA	fwd	β-actin (GeneID: 60)
**b-act as**	CGCAGCTCATTGTAGAAGGTGTGGT	fwd	β-actin (GeneID: 60)
